# Designing Spinel Li_4_Ti_5_O_12_ Electrode as Anode Material for Poly(ethylene)oxide-Based Solid-State Batteries

**DOI:** 10.3390/ma14051213

**Published:** 2021-03-04

**Authors:** Ander Orue Mendizabal, Nuria Gomez, Frédéric Aguesse, Pedro López-Aranguren

**Affiliations:** Center for Cooperative Research on Alternative Energies (CIC energiGUNE), Basque Research and Technology Alliance (BRTA), Parque Tecnológico de Álava, Albert Einstein 48, 01510 Vitoria-Gasteiz, Spain; aorue@cicenergigune.com (A.O.M.); ngomez@cicenergigune.com (N.G.); faguesse@cicenergigune.com (F.A.)

**Keywords:** solid-state battery, polyethylene(oxide), Li_4_Ti_5_O_12_, anode materials

## Abstract

The development of a promising Li metal solid-state battery (SSB) is currently hindered by the instability of Li metal during electrodeposition; which is the main cause of dendrite growth and cell failure at elevated currents. The replacement of Li metal anode by spinel Li_4_Ti_5_O_12_ (LTO) in SSBs would avoid such problems, endowing the battery with its excellent features such as long cycling performance, high safety and easy fabrication. In the present work, we provide an evaluation of the electrochemical properties of poly(ethylene)oxide (PEO)-based solid-state batteries using LTO as the active material. Electrode laminates have been developed and optimized using electronic conductive additives with different morphologies such as carbon black and multiwalled carbon nanotubes. The electrochemical performance of the electrodes was assessed on half-cells using a PEO-based solid electrolyte and a lithium metal anode. The optimized electrodes displayed an enhanced capability rate, delivering 150 mAh g^−1^ at C/2, and a stable lifespan over 140 cycles at C/20 with a capacity retention of 83%. Moreover, postmortem characterization did not evidence any morphological degradation of the components after ageing, highlighting the long-cycling feature of the LTO electrodes. The present results bring out the opportunity to build high-performance solid-state batteries using LTO as anode material.

## 1. Introduction

Li ion batteries (LIBs) provide a unique combination of high energy, power and cyclability that makes them the first choice to power portable electronics and electric vehicles (EVs). In spite of this, the rapidly growing EV industry is imposing highly demanding requirements which are a challenge for a technology that is currently reaching a safety, lifetime and energy density plateau [1]. Within the race towards high-voltage and high-energy density active materials [2], liquid organic electrolytes result in electrochemical stability issues along with safety concerns due to their high volatility and flammability. The replacement of the liquid organic electrolyte in conventional LIBs by solid electrolytes, considered as a solution to high-capacity lithium-metal anodes owing to their mechanical strength, in solid-state batteries (SSBs), offers outstanding high-energy and high-power densities as well as inherent safety [3,4,5,6]. However, far from being a reality, there are still many challenges to overcome on the use of lithium metal anodes for solid-state batteries [7,8]: most of the solid inorganic Li ion conductors are thermodynamically unstable [9] and dendrite growth is still a problem due to the inhomogeneous dissolution and subsequent deposition over cycling [8,10,11,12]. In addition, the continuous stripping and plating of Li metal needs to be fast enough to ensure their performance at an acceptable C-rate.

Among the wide range of solid electrolytes, organic solid polymer electrolytes have attracted widespread attention over their inorganic electrolyte competitors due to relevant advantages such a low density, a lower interfacial resistance between the components, ease of processing and excellent mechanical properties [13,14,15,16]. However, batteries including dry solid polymer electrolytes for which poly(ethylene)oxide (PEO) with conductive lithium salts is the forefront material need to operate at temperatures above 60 °C due to their low ionic conductivity (10^−6^ S cm^−1^) and Li^+^ transference number (0.3) at room temperature, which affects the rate capability of the SSB. Nevertheless, solid-state PEO-based batteries have shown very promising performances [17]; indeed, a LiFePO_4_ PEO-based cell with lithium metal anode is commercially available for EVs [18]. In spite of the limited electrochemical stability of PEO (<3.8 V vs. Li/Li^+^) [6], successful combination with high-voltage active materials such as LiCoO_2_ [19,20,21], Li(Ni_x_Co_y_Mn_1−x−y_)O_2_ [22,23] and LiNi_0.5_Mn_1.5_O_4_ [24] foresees a promising future for this chemistry, but still with important limitations on the anode side. The replacement of Li metal negative electrode by the spinel Li_4_Ti_5_O_12_ (LTO) negative material would avoid stripping–plating issues, dendrite growth and will ensure high performance devices with improved safety. LTO negative material delivers 175 mAh g^−1^ at an operating potential of 1.5 V vs. Li/Li^+^. This material exhibits an exceptional electrochemical stability and is one of the most appealing negative materials to power lithium ion batteries in applications that require high currents, a long cycle life and high efficiency [25]. Moreover, matching LTO with positive materials such as LiFePO_4_ or LiMnO_2_ [26] can still offer competitive energy density of 100 Wh kg^−1^–270 Wh L^−1^ and 150 Wh kg^−1^–375 Wh L^−1^, respectively, at reasonable areal loadings of 3.5 mAh cm^−2^.

In SSBs, the heterogeneous nature of positive and/or negative electrodes results in various resistive interfaces and interphases of different nature and quality, jeopardizing the smooth performance of the cell, owing to multiple sources of impedance, by decreasing the capacity, increasing the polarization or inhibiting fast rate cycling [27,28]. Therefore, an important challenge of solid-state batteries is thus to decrease such resistive sources by improving ionic and electronic conductivity. A robust electronic network at the electrode is essential to enable the transport of electrons between particles and with the current collector, ensuring fast kinetics of (de)lithiation reactions [29]. Additives with fibrous morphology like nanostructured multiwalled carbon nanotubes (CNTs) show a high intrinsic electronic conductivity along the carbon fiber axis, thus providing a larger amount of electron-transport pathways for electroactive particles [30]. In this regard, CNTs have been studied for battery applications due to their excellent electronic conductivity, large specific surface area, high mesoporosity and good electrolyte accessibility [30,31,32,33].

In the present work, we report for the first time the electrochemical performance of LTO electrodes with a PEO-based electrolyte. High performance electrodes have been prepared and optimized from a careful selection of electronic conductive additives and electrode engineering, featuring the advantages of LTO as anode material for solid-state batteries [34]. The electrochemical properties of the as-prepared electrodes have been evaluated in half-cells with a PEO-based electrolyte and a Li metal anode at 70 °C. The type of electronic conductive additive has proven to have a strong impact on the rate capability of the electrodes. The partial replacement of carbon black C65 by CNTs led to improved electrodes showing an enhanced remarkable rate capability up to 4 C, in addition to a long-cycle life with low ageing of the components after 140 cycles at C/20.

## 2. Materials and Methods

### 2.1. Preparation of Li Metal–LTO Solid-State Cells

Li metal solid-state cells were assembled for their electrochemical characterization. The full cells were composed of 3 components: the Li metal anode; the solid polymer electrolyte (SPE), used as separator; and the LTO cathode electrode, containing a catholyte of the same composition as the SPE.

The SPE was prepared from a mixture of bis(trifluoromethane) sulfonamide lithium salt (LiTFSI, Sigma Aldrich, St. Louis, MO, USA, 9.95%) and polyethylene oxide (PEO, Sigma Aldrich, St. Louis, MO, USA, M_w_ = 5 × 10^6^). To avoid any trace of water, PEO and LiTFSI were dried at 50 and 80 °C, respectively, in a vacuum oven for 12 h before being transferred to an Argon (Ar) glove box. To prepare the membrane, PEO and LiTFSI were stirred in anhydrous acetonitrile (ACN, Sigma Aldrich, St. Louis, MO, USA, 99.8%) for 12 h. The salt concentration was fixed at the optimized molar ratio of –CH_2_CH_2_O-(EO)/Li = 20:1. The as-milled slurry was casted onto a Mylar sheet using a Hohsen MC-20 Minicoater with a blade gap of 2300 μm and a speed of 10 mm s^−1^. Finally, the casted membrane was dried at 50 °C for 12 h under dynamic vacuum.

Two LTO electrodes laminates were prepared similarly using a carbon-coated Li_4_Ti_5_O_12_ (NEI Corporation, Somerset, NJ, USA, 3–5 µm particle size), and carbon black (C65, Imerys, Paris, France) as conductive agent (CA). In one of the electrodes, part of C65 was replaced by multiwalled CNTs (Sigma-Aldrich, St. Louis, MO, USA). The ratio C65:CNTs was fixed to 100:0 and 50:50 for the electrodes labelled as LTO-C65 and LTO-C65/CNTs, respectively, to follow the influence of the CNTs. The starting materials were dried at 80 °C for 12 h under vacuum and then mixed with an IKA^®^ ULTRA-TURRAX^®^ disperser at a speed of 16,000 rpm for 15 min. The resulting slurry was casted onto aluminum foil (thickness of 20 μm) at a speed of 2.5 mm s^−1^. Finally, the laminate was dried at 80 °C for 12 h under dynamic vacuum. The electrode sheets display an active material loading of 3 mg cm^−2^ (capacity of the electrode is 0.55 mAh cm^−2^). The composition of the electrodes of LTO: CA: SPE was fixed to 75:10:15 wt.%.

### 2.2. Microscopical Features of the Electrodes

The morphological characterization of the electrodes was conducted via scanning electron microscopy (SEM, Thermofisher, Waltham, MA, USA) with a FEI Quanta 200F SEM-EDX station (ScopeM, Zurich, Switzerland) (with the acceleration voltage ranging between 20 and 30 keV collecting either secondary or backscattered electrons). The electrodes were hardened using liquid nitrogen and then cut with a blade in order to obtain a sharp cross section. The composition was determined by energy dispersive X-ray spectroscopy (EDX) using an Oxford Instrument detector (Oxford Instruments plc., Abingdon, UK). For the postcycling studies, the cells were carefully disassembled under an Ar filled glovebox. Then, cross-sectioning of the solid-state cell was performed via ion milling with an IM4000 equipment (Hitachi, Krefeld, Germany) operating in cryostat mode to avoid heating of the polymer.

### 2.3. Electrochemical Evaluation of the Electrodes

The electrochemical characterization of the cells was performed in CR2032 coin cells. Full cells were assembled with a 16 mm diameter disk of the membrane sandwiched between disks of 12 and 14 mm diameter of the LTO laminate and a 500 µm thickness Li metal, respectively.

Electrochemical Impedance Spectroscopy (EIS) and the rate capability tests of the full cells were realized on a VMP3 (BioLogic, Seyssinet-Pariset, France) at 70 °C. Prior measurements the cells were conditioned at 70 °C during 24 h in order to minimize the interfacial resistances of the cell. EIS was performed at frequencies ranging from 0.01 MHz to 1 Hz and 50 mV polarization amplitude. The rate capability of the cells was evaluated between 1.0–3.0 V vs. Li/Li^+^ at the following C-rates: C/20, C/10, C/5, C/2, 1 C, 2 C, 4 C for 5 cycles each step and back to C/10. The charging time step was set to a 10% higher than the corresponding one at each current.

## 3. Results

### 3.1. Microscopical Features and Composition of the Electrode Laminates

**Figure 1:**
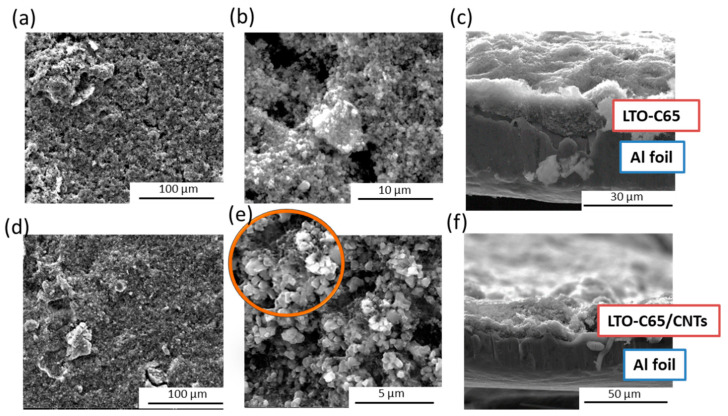
(**a**–**c**) Microscopical features of LTO-C65 and (**d**–**f**) LTO-C65/CNTs electrodes. Micrographs (**a**,**b**,**d**,**e**) correspond to top-views at different magnifications and (**c**,**f**) to cross-sections of the laminates.

Figure 1 shows the top-view and cross-section micrographs of the LTO electrodes with the electronic conductive additive C65 (LTO-C65) and the mixture of C65 and CNTs (LTO-C65/CNTs). Top views confirmed the smooth surface with sparse pores for both electrodes, in agreement with the homogenous distribution of the micron-sized LTO particles observed at higher magnifications. A close inspection on the LTO-C65/CNTs revealed the elongated morphology of the CNTs randomly arranged at the electrode. The cross-section of both electrodes, casted on the aluminum substrate and exhibiting an active material loading of 0.55 mAh cm^−2^, showed a thickness of ~30 μm.

### 3.2. Electrochemical Performance of the Full Cells

#### 3.2.1. Rate Capability

**Figure 2:**
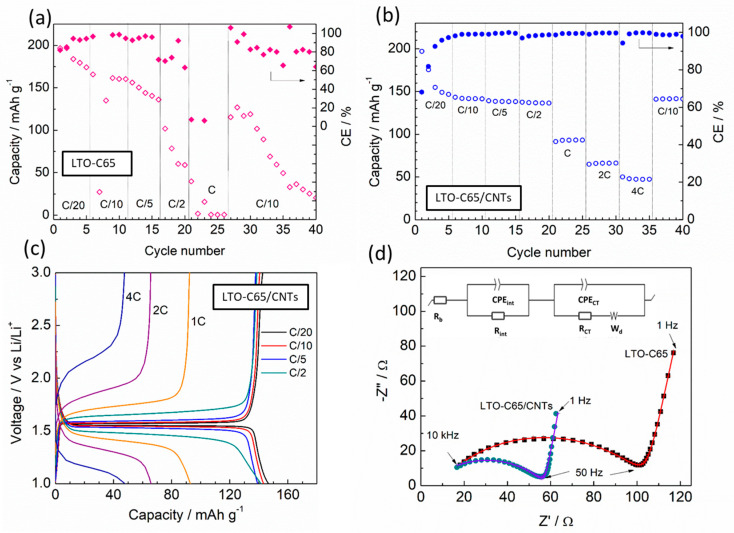
Rate capability of (**a**) LTO-C65 and (**b**) LTO-C65/CNTs half-cells with PEO-LiTFSI polymer electrolyte and Li metal anode measured at 70 °C. (**c**) Charge–discharge voltage profile of the LTO-C65/CNTs cell from C/20 to 4C. (**d**) Nyquist plots (dots) and equivalent circuit (lines) of the LTO-C65 and LTO-C65/CNTs cells after conditioning 24 h at 70 °C.

Figure 2 compares the rate capability behavior at 70 °C of LTO-C65 and LTO-C65/CNTs as solid-state cells using a PEO-LiTFSI electrolyte and Li metal anodes. During the initial cycles, the LTO-C65 cell delivers an initial discharge capacity of ca. 200 mAh g^−1^ (Figure 2a), exceeding the theoretical capacity of spinel LTO, which fades rapidly upon cycling. After a few cycles, the cell delivers 150 mAh g^−1^ at C/20 and decreases down to ca. 130 mAh g^−1^ at C/5. The poor performance of this electrode is evidenced by the remarkable capacity loss as well as the low and unstable coulombic efficiency (CE), with charging steps sometimes reaching the 10% time limitation. At higher currents of 1 C, the LTO-C65 cell experiences a severe fading with no signs of red-ox response. However, the cell is able to recover 125 mAh g^−1^ at C/10 for a few cycles, although the capacity retention drops down at the 40th cycle, evidencing the end-of-life for this cell. The rate capability of the LTO-C65/CNTs electrode (Figure 2b) evidences at first sight the enhanced electrochemical performance of the cell, promoted by the addition of CNTs. The cell shows a superior behavior delivering ca. 150 mAh g^−1^ up to C/2 and a remarkable discharge capacity at faster rates: at 1 C, the cell displays 90 mAh g^−1^ and at 4 C (15 min of charge and discharge) delivers ca. 50 mAh g^−1^, with a CE remaining as high as 99.5%. Moreover, the cell is able to recover 150 mAh g^−1^ at C/10 with no signs of fading after 40 cycles, thus proving its excellent stability and reversibility. Figure 2c shows the charge–discharge voltage profiles for the optimized LTO-C65/CNTs electrode from C/20 to 4 C. As observed, the red-ox plateaus between C/20 and C/2 remain flat, with a polarization below 60 mV. However, above C/2 the plateau turns into a sloppy profile with a polarization increasing above 250 mV, which could be ascribed to the insufficient ionic conductivity of the solid electrolyte leading to kinetic limitations on the Li-ion transport in agreement to the low capacity observed at fast rates. The superior performance of the electrode including CNTs is supported by the impedance spectra shown in Figure 2d. The Nyquist plot of the cells with both electrodes shows a well-defined semicircle at high frequencies (0.01 MHz–100 Hz) followed by a spike from 100 Hz to 1 Hz. The profiles and resistance of the full cells are similar to previous studies of PEO-based batteries [17,22]. The EIS plots have been well fitted using an equivalent circuit (Ec) (Figure 2d) [35] which distinguishes the ohmic resistance of the bulk solid electrolyte (R_b_), the resistance of the electrode interfaces (R_int_) i.e.,: active material/polymer electrolye resistance, electrode/solid electrolyte interface, the charge transfer resistance for electrochemical reactions (R_ct_), the constant phase elements (CPE) attributed to the double-layer capacitance of the porous electrode and a finite length Warburg contribution (W_d_) representing diffusion processes [36]. Values of the resistance from the different elements are summarized in Table 1. A similar bulk resistance of LTO-C65 and LTO-C65/CNTs was found to be 8 and 3 Ω, respectively. Such slight difference might be attributable to the higher conductivity of the LTO-C65/CNTs catholyte by an enhanced amorphization by local modification of PEO chains from crystalline to disordered arrangements promoted by the CNTs [37]. Both cells display an interfacial electrode resistance, with a capacitance value of ca. 10^−6^ F, which is 50% lower for the CNTs electrode, evidencing that the addition of CNTs decreases the interfacial resistances at electrode level, in agreement with the superior performance of this electrode. A very low resistance below 1 Ω with capacitances of 10^−3^ F was found for both full-cells and was ascribed to electrochemical reactions.

#### 3.2.2. Long-Term Cycling and Postmortem Analysis

**Figure 3:**
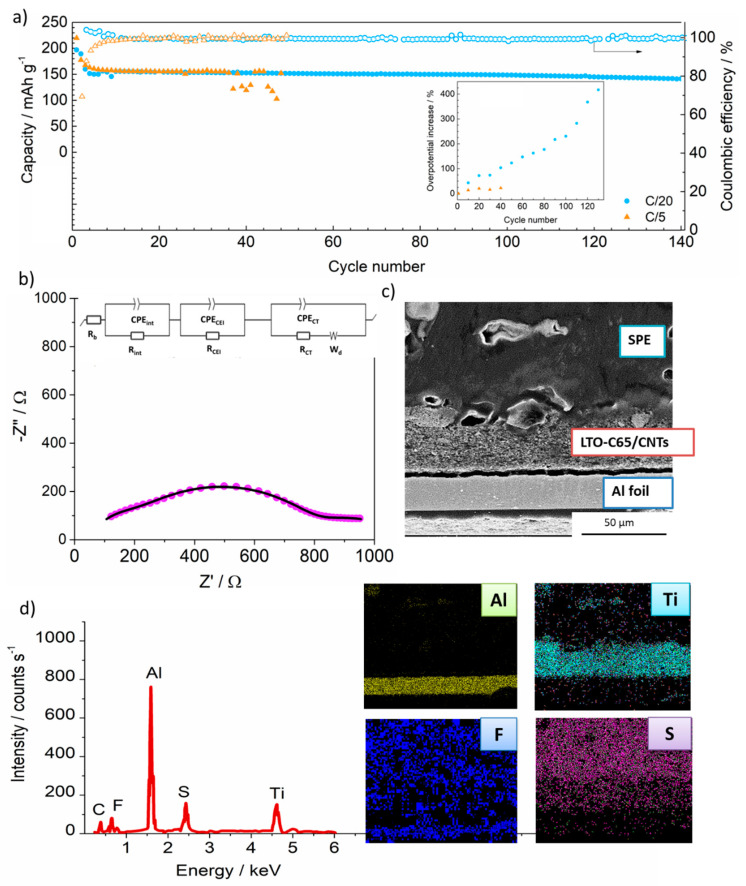
(**a**) Discharge capacity and coulombic efficiency (CE) of the long-term cycling of the LTO-C65/CNTs electrode at C/20 (blue circles) and at C/5 (orange triangles). (**b**) Nyquist-plot of the cell after 140 cycles at C/20 (pink circles) and fitting results of the equivalent circuit (black line) schematized at the top. (**c**) Cross-sectioned micrograph of the bilayer including the SPE and the electrode after 140 cycles and (**d**) EDX mapping of the bilayer thickness area.

The optimized electrode, LTO-C65/CNTs, was subjected to long-term tests in order to determine the lifespan of the cell cycled under high and low current density. The performance of the cell at C/5 and C/20 is shown in Figure 3a. At C/5, the cell shows 100% of the capacity retention and CE for 35 cycles, after which the cell starts malfunctioning, as evidenced by the poor and dispersed capacity. However, at lower currents of C/20 the cell shows a stable cycling with 100% CE and a remarkable capacity of 150 mAh g^−1^ after 140 cycles. The long-life cycle of the cell operating at C/20 in contrast to C/5 points out a different degradation mechanism occurring at higher currents.

We conducted electrochemical and physicochemical characterization after the cycling of LTO-C65/CNTs cell in order to provide insights on the ageing and ultimate failure of the components. An EIS of the cell was obtained at the end of the 140th cycle after cycling at C/20. The Nyquist plot (Figure 3b) evidences an increase in the overall resistance above 1000 Ω, compared to the low resistance before cycling (Figure 2d and Table 1). Several resistances have been identified for this cell after cycling. On the one side, the bulk resistance increases from 3 to 15 Ω after cycling, likely due to a slightly lower conductivity of the electrolyte arising from the degradation of the polymer and/or LiTFSI salt. Besides, the interfacial resistances at the electrodes (R_int_) increase up to 298 Ω. Such large resistance arises from physicochemical processes occurring at the SPE/Li metal interface or internal interfaces at the LTO electrode. An additional resistance appears after cycling and has been ascribed to the formation of a cathode solid electrolyte interface resistance (R_CEI_), which could explain the irreversible capacity observed during the initial cycles (Figure 1a,b and Figure 2a) [35]. Despite such large resistive CEI, the high discharge capacity and the high coulombic efficiency attained after 140 cycles evidence the positive contribution of this stable interface.

The evolution of the overpotential of the cell over cycling has been plotted in the inset of Figure 3a. At C/20 the overpotential increases from 25 mV to 140 mV after 140 cycles, which corresponds to an increase of 400%. At C/5, the initial overpotential, 70 mV, is much higher than that at C/20, although after 40 cycles the increase remains below 25%. These results may indicate that cycling at lower currents induces a slow degradation of the cell interfaces, evidenced by the large resistance as well as the high overpotential of the cell. However, at higher currents the failure of the cell is probably induced by the formation of dendrites [10], as observed from the sudden loss of capacity at C/5. The high current density is prone to favor dendritic Li metal during Li electrodeposition. The morphology and corresponding elemental distribution of the interfaces were examined by the cross-sectional SEM and elemental mapping, respectively [38]. During the disassembly of the full cell, the Li metal was detached from the solid electrolyte. Then, the bilayer of electrolyte and electrode was cross sectioned by ion milling. The cross-section from Figure 3c confirms the retention of the structural integrity of all the aged components as well as the contact at the solid electrolyte—electrode interface, in spite of more than 5600 h of cycling (140 cycles). Neither cracks nor voids are observed along the electrode thickness. The detachment of the electrode from the Al substrate of ca. 3 μm length may indicate some loss of binding properties over cycling, which would also explain the increase of R_int_, although it may arise from the manipulation during the preparation of the sample. Figure 3d shows the examination by EDX mapping of the squared scan across the bilayer thickness. The chemical composition analysis indicates the presence of Al from the substrate, Ti from the LTO active material and F and S species from the Li salt (LiTFSI). No segregation or diffusion of the elements across the thickness is observed and the uniform distribution of titanium is in agreement with the homogenous dispersion of the particles observed on the SEM images (Figure 1) from the pristine electrodes.

## 4. Discussion on the Electrode Design for Solid-State Batteries

The present work highlights the promising prospects of LTO as anode material for solid-state PEO batteries. In addition, it demonstrates the importance of a careful electrode design, with special attention on the electronic additives [34]. Carbon nanotubes provide an improved and robust electronic conductivity network at the electrode, thereby overcoming important limitations of solid-state batteries. The partial replacement of C65 by CNTs effectively enhances the power capability as well as the long cycling life of the LTO anodes. This is confirmed by the high rate capability up to C/2 as well as the stable cycling at C/20 during 140 cycles, delivering 150 mAh g^−1^, thus being particularly suitable for stationary applications with guaranteed high power peak demands. Cycling at a rate of C/5 leads to failure of the cell after 40 h, probably caused by dendrite formation promoted by the Li metal anode. However, cycling at lower currents of C/20 induces a slow degradation of the cell interfaces, ensuring a long cycling life of the cell. Further research coupling LTO electrodes with high-voltage positive materials is envisaged to take this technology to the next level. We demonstrate that the integration of LTO electrode in solid-state batteries is valuable and shows very promising performance.

## Figures and Tables

**Table 1:** Resistances of the different components obtained from the Li metal full cells assembled with the two electrodes: ionic bulk conductivity (R_b_), electrode interfaces (R_int_), cathode solid electrolyte interface resistance (R_CEI_) and charge transfer resistance for electrochemical reactions (R_ct_). The resistances are calculated from the equivalent circuit before cycling of the cells and for the LTO-C65/CNTs cell after 140 cycles at C/20. Values are given in Ohms.

Sample	R_b_	R_int_	R_CEI_	R_CT_
LTO-C65	8	97	-	<1
LTO-C65/CNTs	3	54	-	<1
Cycled (C/20)-LTO-C65/CNTs	15	298	394	307

## Data Availability

Data is contained within the article.
